# Improvement in low back movement control, decreased pain and disability, resulting from specific exercise intervention

**DOI:** 10.1186/1758-2555-2-11

**Published:** 2010-04-23

**Authors:** Hannu Luomajoki, Jan Kool, Eling D de Bruin, Olavi Airaksinen

**Affiliations:** 1 Physiotherapie Reinach, Private Practice, 5734 Reinach, Switzerland; 2 University of Kuopio, Medical Faculty, Sport Medicin, Kuopio, Finland; 3 Institute of Physiotherapy, Department of Health, Zürich University of Applied Sciences, Winterthur, Switzerland; 4 Institute of Human Movement Sciences and Sport, ETH Zurich, Zurich, Switzerland; 5 Department of Physical and Rehabilitation Medicine, University Hospital of Kuopio, Finland

## Abstract

**Background:**

The study was conducted to assess whether patient-specific functional impairment and experienced daily disability improved after treatment to address active movement control of the low back.

**Method:**

A prospective study was carried out in two outpatient physiotherapy practices in the German-speaking part of Switzerland. 38 patients (17 males and 21 females) suffering from non-specific low back pain (NSLBP) and movement control impairment were treated. The study participants had an average age of 45 ± 13 years, an average height of 170 ± 8 cm and an average weight of 73 ± 15 kg. Patients were assessed prior and post treatment. Treatment was aimed at improving movement control of the lumbar spine, pain and disability. Six physiotherapists treated each patient on average nine times (SD 4.6). Treatment effects were evaluated using a set of six movement control tests (MCT), patient-specific functional pain scores (PSFS) and a Roland and Morris disability questionnaire (RMQ). Means, standard deviations, confidence intervals and paired t-tests were calculated. The effect size (d) was based on the change between t1 (time prior intervention) and t2 (time post intervention) using a significance level of p < 0.05, with d > 0.8 being considered a large effect. Power calculations were performed for type I & II error estimation.

**Results:**

Movement control (MCT) showed a 59% improvement from 3.2 (max 6) to 1.3 positive tests (d = 1.3, p < 0.001), complaints (PSFS) decreased 41% from 5.9 points (max 10) to 3.5 (d = 1.3, p < 0.001), and disability (RMQ) decreased 43% from 8.9 to 5.1 points (d = 1.0, p < 0.001).

**Conclusions:**

The results of this controlled case series study, based on prior and post intervention, showed that movement control, patient specific functional complaints and disability improved significantly following specific individual exercise programs, performed with physiotherapeutic intervention. The results obtained warrant performance of a randomized controlled trial (RCT) to substantiate our findings.

## Background

Low Back Pain (LBP) is a huge medical and financial problem in the industrialized world [1,2]. A specific diagnosis of LBP is only possible in 15% of patients. In the majority of cases, LBP is non-specific (NSLBP) [1,3,4]. In 80-90% of individuals suffering an acute episode of LBP the prognosis for rapid improvement in pain and disability, a return to work within one month, followed by further slight improvements for up to three months [5,6], is good. However, a small proportion of patients (10-20%) develop chronic NSLBP, usually defined as pain persisting for longer than 3 months [2,7]. Up to 70% of those who initially improve experience repeated fluctuating pain episodes [5]. The treatment of these patients represents one of the biggest challenges of modern healthcare. To improve assessment and treatment regimes the identification of different subgroups of patients with NSLBP and the development of tailored, more efficient treatments has high priority [2]. Recent research has developed clinical tests to identify a subgroup of patients with NSLBP with impaired movement control (MC) [8-15].

Movement impairment syndromes and the detection of disordered movement, or pathokinesiology, is a key competence of physiotherapy [13,14]. Pathokinesiologic movement patterns in the lumbar spine have been investigated and described [8-10,12,16-18], resulting in the publication of both reliability and validation studies of the examination procedures used [11,12,18-21]. However, no clear evidence exists as to whether improvement in movement control can also lead to decreased pain and experienced daily disability in patients with NSLBP.

The underlying hypothesis is that due to poor movement control (MC) of the back, a person is unknowingly damaging him- or herself through faulty movement patterns. O'Sullivan describes these back pain patients not as pain avoiders but, as pain provocateurs [10]. Relative flexibility theory [13,22] suggests that movement occurs through the pathway of least effort, e.g. if the hip movement is stiff relative to that of the low back, then the range of back movement is greater and would lead to a back pain problem related to the direction of that particular movement. Widely used synonyms for movement impairment syndromes are motor control dysfunctions [14,15] and MC impairment [8,10].

In earlier studies, we showed that a set of six tests was able to reliably assess the movement control ability of the low back [20]. Other authors have reported similar results [19,23,24]. Comprehensive reviews of treatments of low back pain show clearly that exercise is the best conservative treatment of the low back pain condition [2,25-27]. However, the results regarding the most appropriate type of exercise are controversial. Most studies failed to consider the subgroups of NSLBP of participating patients [28,29]. A small number of studies that specifically address subgroups showed positive results where therapy was targeted at the specific problems of the subgroup [30,31]. According to the European guidelines for the management of chronic non-specific low back pain [2], high quality clinical trials are needed to determine the effectiveness of specific interventions aimed at specific target groups.

Movement control impairment syndrome is a clear subgroup of NSLBP. The reliability and validity of tests to diagnose this syndrome have been shown to be acceptable [20,32]. This study asked whether it is possible to improve movement control and, if so, whether movement control improvement is accompanied with a reduction in patient specific complaints, pain and disability. The aim of this study, therefore, was to determine whether improvement in movement control of the low back concurrently decreases patient-specific functional impairment and experienced daily disability. Patients with non-specific low back pain (NSLBP) and impaired movement control were tested prior and post a course of treatment, which was aimed at improving movement control of the lumbar spine.

## Methods

### Study design

A prospective study was carried out in two private outpatient physiotherapy practices in Canton Aargau, Switzerland. Data collection took place between April and September 2007. The research was approved by the ethics committee of the government health authorities of Canton Aargau, Switzerland. Written, informed consent was obtained from all patients in advance of the study.

### Study subjects

Selection of the subjects was conducted by physiotherapists in the two clinics between the above dates. The criterion for inclusion of patients was that they suffered from non-specific low back pain (NSLBP). This included local LBP, or radiating pain, but without neurological findings (muscle weakness, loss of sensibility or reflexes) [1]. The exclusion criteria were serious pathologies, such as unhealed fractures, tumours, acute trauma or serious illnesses. A measurement criterion required that patients have at least 3 out of 6 MC tests positive [32,33]. Further assessment measures included the Patient-Specific Functional Scale (PSFS) [34] and Roland Morris disability questionnaire (RMQ) [35,36]. To avoid ceiling effects, they needed to have at least 3 out of 10 as a mean value on the PSFS and 5 out of 24 points on RMQ. The patients also had to be able to understand instructions in German. Following application of the above criteria, suitable patients were selected, explained the aims of the study and asked to participate.

### Examination

A test battery consisting of six active movement control tests, based on descriptions by Sahrmann and O'Sullivan [9,10,13,19,22], and which has been published in our earlier papers [20] {Luomajoki, 2008 #379} was applied. In an earlier study, the reliability of these tests was shown to be good [33]. Since the MC tests are direction specific, a battery of tests is required for the analysis. Subjects were tested in a standardized manner by performing the complete set of tests. Each positive test scored one point, i.e. 3/6 meaning the subject performed 3 tests wrongly. The assessors were physiotherapists trained in the assessment of MC dysfunctions. The criteria were discussed and typical patterns of dysfunctions were presented. In addition, the Roland Morris questionnaire (RMQ) [36] and patient-specific functional score (PSFS) [34] were assessed at the beginning and at the end of the set of tests. Both questionnaires have been shown to be valid and reliable [37-39]. Over the last 20 years RMQ is the most used measurement tool for LBP. PSFS has been shown to have very good responsiveness to improvement in LBP [40]. Six physiotherapists participated in examining and treating the patients. Following treatment, a blinded assessor reassessed the patients' movement control.

### Intervention

The physiotherapists treating the patients were instructed to improve the movement control ability of the subjects on an individual basis, according to published prescriptions [9,13-16,18,22]. Each therapist could decide himself how best to reach the set goals with his individual patients. Patients were also provided with an individual exercise program which they could do at home. Six physiotherapists treated each patient on average nine times (SD 4.6).

The physiotherapists treating the patients had an average of seven years working experience and had studied manual therapy at post graduate level for a minimum of four weeks. All were stationed in private physiotherapy practices.

### Analysis

Movement control was assessed prior and post the treatment series. Percentages of mean change and point values were calculated, as well as standard deviations and confidence intervals. Paired t-tests, for parametric data, and Wilcoxon rank tests, for non-parametric data, were performed for intragroup changes between t1 and t2. The effect sizes (d) between t1 and t2 were calculated, with d > 0.8 being considered a large effect, and the significance level set by p < 0.05. The data were analyzed with SPSS 14.0 for Windows. Power analysis for sample size calculation for a RCT was performed on the acquired data.

## Results

Initially 96 LBP patients were screened for impairments in their movement control ability. 50 patients, measuring at least 3/6 tests positive, were asked to participate. Out of these, a total of nine patients were excluded: six due to ceiling effects on RMQ or PSFS, two because of a neurological nerve root condition, and one subject as a result of having pain levels too high to be able to perform the movement tests. Of the 41 remaining included patients three (7%) were not available for the follow up assessment. (The condition of one deteriorated, resulting in discontinuation of therapy, and two subjects did not appear, explaining by telephone that they had no more back pain and did not want to come for another examination). The status of 38 patients was assessed at t2. Mean age of the participants was 45 years (SD ± 13), Height 170 cm (± 8), Weight 73 kg (± 15), RMQ 8.9 (± 4.7), mean PSFS 5.7 (± 1.5), MCT score 3.2 (± 1.2). Of the participants, the majority 28 people (74%) were working, 5 persons (13%), on disability allowance, 3 people (8%) were students and 2 people (5%) retirees. Average duration of their LBP was 18 (6) months.

**Figure 1:**
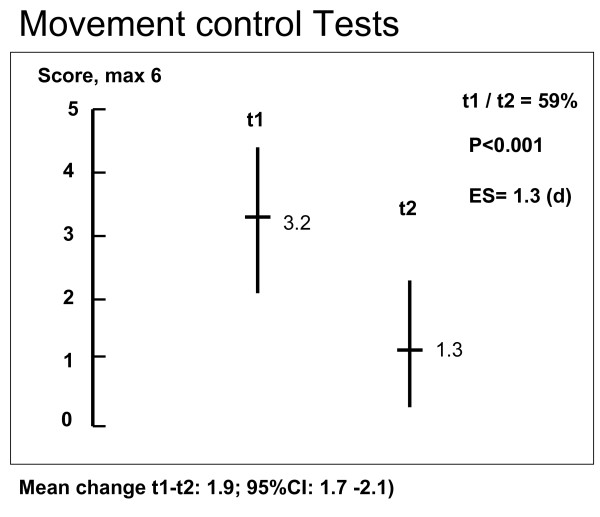
**Results of measurements t1 and t2 for Movement Control Test battery (MCT)**.

**Figure 2:**
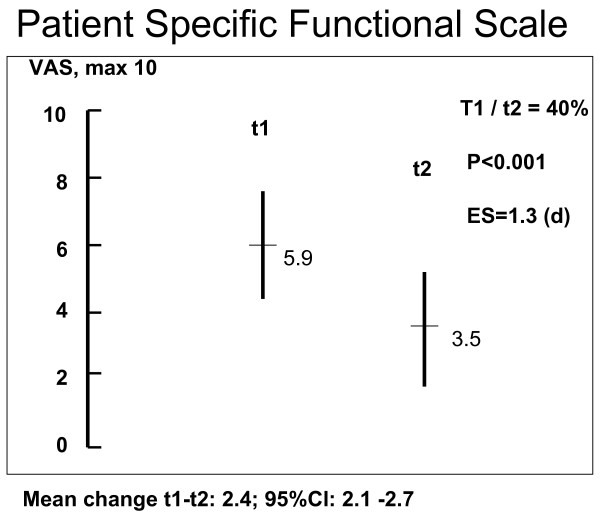
**Results of measurements t1 and t2 for Patient Specific Functional Score (PSFS)**.

**Figure 3:**
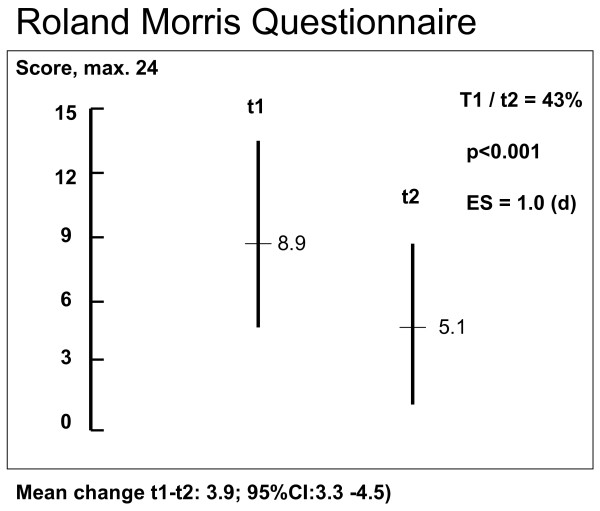
**Results of measurements t1 and t2 for Roland Morris Disability Questionnaire (RMQ)**.

Table 1 and Figures 1, 2 and 3 show the results at t1 and t2, respectively. The results of positive movement control tests improved 59% from 3.2 (max 6) to 1.3 (d = 1.3, p < 0.001), complaints (PSFS, max 10) decreased 41% from 5.9 points to 3.5 (d = 1.3, p < 0.001) and disability (RMQ, max 24) decreased 43% from 8.9 points to 5.1 (d = 1.0, p < 0.001). Point values and SDs for the change between t1 and t2 were: in MCT 1.9 (1.5), PSFS 2.4 (1.9) and RMQ 3.9 (3.9).

**Table 1:** Change in movement control (6 tests) and disability (Roland Morris Disability Questionnaire- RMQ) and patient specific functional scale-PSFS.

N = 38	t1	t2	Change t1 to t2 (SD)	ES (d)	Sig (paired t-test/Wilcoxon signed rank test *)
MC N = Positive tests (SD)	3.2 (1.2)	1.3 (1.2)	1.9 (1.5)	1.3	< 0.001
RMQ mean (SD)	8.9 (4.8)	5.1. (3.7)	3.9 (3.9)	1.0	< 0.001*
PSFS mean (SD)	5.9 (1.6)	3.5 (2.0)	2.4 (1.9)	1.3	< 0.001*

Effect Size: d < 0.2 small, *0.5 moderate*, > 0.8 large.

Based on our results, we performed sample size calculations for a planned randomized controlled trial (RCT). We used a power of 0.9 and alpha was set at 0.05. In order to be able to detect a PSFS 1.3 (SD of 1.8) point difference in complaints between an experimental and control group, 40 patients in each group would be needed to detect a significant intergroup difference in the number of positive movement control tests. Detecting a benefit of 1.9 with a SD of 1.5, 48 patients would be needed. If disability, assessed with the RMQ, was chosen as the main outcome, 81 subjects in each group would be required to show a significant intergroup difference of 2 points (clinically meaningful change; [41]) with a SD of 3.9. According to O'Sullivan's estimation, up to 30% of patients with LBP mainly have movement control impairment. So, we can conclude that for a RCT with adequate power and 100 patients, 300 patients would have to be initially assessed for eligibility.

## Discussion

To the best of our knowledge, this is the first study to evaluate a series of cases on movement control ability following physiotherapy treatment based on published prescriptions. The observed improvement in MC was accompanied by decreased functional experienced pain and disability in patients with NSLBP pain. The biggest effect was shown in the improvement of movement control ability.

There is evidence to indicate that patients with movement control deficits are an important subgroup of LBP and that they may benefit from specific exercises [9,13-16,18,22]. Only about 10-15% of patients can be diagnosed with specific LBP [1,2]. O'Sullivan [9,10] developed a classification system of LBP. The first distinction is between centrally evoked and peripherally evoked LBP. The centrally evoked pain is associated with psychological factors, such as fear avoidance, catastrophizing or depressive mood (approximately 30% of LBP patients). The peripherally evoked LBP is mechanically caused and includes movement impairment and movement control impairment (each approximately 30%). Patients with movement impairment have a painful restriction of movement. Patients with movement control impairment have complaints in certain positions, such as sitting, standing or in twisted positions. Movement control impairment is direction specific, either provoked by flexion, extension, rotation or multidirectional movements. According to O'Sullivan, up to one third of patients with LBP are estimated to have movement control impairment. In a Delphi study of American physical therapists, who were Orthopaedic Clinical Specialists or Fellows of the American Academy of Orthopaedic Manual Physical Therapists (N = 168) [42], 88% of the specialized therapists considered abnormal movement patterns as the main finding in clinical instability of the low back.

Large reviews conclude that there is strong evidence for the effectiveness of exercise as a treatment for LBP [2,25,43]. For instance, activity and general exercise therapy improves pain and disability and reduces the number of sick days in patients with non-specific chronic low back pain [44-46]. Nevertheless, it is not clear what kind of exercises should be used. Stabilizing exercises are a popular treatment option, yet reviews of these conclude [47,48] that the outcome of specific stabilization and motor control exercises are not more effective than general exercise programs. However, previous studies paid little attention to the selection of patients to receive specific, individual movement control exercise or general exercise. This may explain the inconclusive results. Positive studies involved defined clinical subgroups. Benefits of specific exercises were demonstrated in other subgroups of patients with LBP. Specific stabilizing exercise is more effective than general exercise in post partum women with pelvic instability [49] and patients with spondylolysthesis [30]. Brennan et al (2006) showed that the outcomes are better if patients receive treatment adapted to their clinical presentation [50]. Treatment options in this study were manipulation, specific individual movement control exercises during a four week intervention. Therapies matched to the patients' clinical problems were more effective in the short and long term.

So far, for the effectiveness of exercises based on specific movement control findings in the low back, three case studies have been published [8,16,22]. Maluf et al. (2000) reported on a single patient who had rotation with extension movement control dysfunction. The 55-year old lady was treated 8 times over 3 months and the Oswestry disability Index reduced from 43% at the beginning to 12% at the 3 month follow up. Van Dillen et al. (2005) treated a 22-year old man with rotation with flexion syndrome who showed an improvement in the Ostwestry Index from 16% to 6% after 4 visits in therapy and then to 4% (follow up 1 year). Visual Analog Scale pain assessment (VAS) decreased from 4/10 to 1/10 after the last visit and to 0/10 after 1 year follow up. Dankaerts et al [8] treated a 37-year old female with flexion control dysfunction and they reported pain freedom after 8 treatment sessions within 14 weeks and reduction of the Oswestry from 34% pre-intervention to 14% post-intervention and 2% in 6 months follow up. These independent single-case studies formed the justification for our study to evaluate movement control exercises in a subgroup of patients with non-specific LBP and impaired movement control. Data from this study might now be used to estimate the required sample size of a future RCT.

Although the results of well-designed observational studies (with either a cohort or a case-control design) do not necessarily systematically overestimate the magnitude of the effects of treatment as compared with those in randomized, controlled trials on the same topic [51], we consider the lack of a control group as a limitation of our study. Therefore, we cannot at present draw conclusions as to how patients would have improved without treatment. In addition, no follow up examinations were conducted. Results were assessed directly after intervention only. It is a possibility that improvements could have vanished relatively quickly following the treatment series. However, in the independent single-case studies the patients showed further improvement in the follow up time period. Our patients were mostly subacute with 73% of them still working. This means that our population might not be typical of patients being at great risk of chronification. There is strong evidence that psycho social issues [52], such as fear avoidance [1,53,54] or catastropizing, are the most pertinent factors leading to chronicity, yet we did not measure any of these properties. Furthermore, we did not subclassify the patients beyond that they should have at least 3/6 tests positive in the MCT battery and were not allowed to show ceiling effects for the other outcome measures. It is clear that patients' clinical behaviour should also match in their movement impairment group. That is also a possible drawback of our study since, although the patients did have movement control deficits, we cannot say whether this was the cause of their back problems. However, with a clearer classification, as suggested by Dankaerts (2006) and Vibe Fersum (2008) and O'Sullivan (2005), even better results could be expected.

The treating physiotherapists were stationed in private physiotherapy practices. This was important, because up to 70% of physiotherapists in Switzerland work in these institutions. We wanted to reflect current practice and try to be as pragmatic as possible in management of the patients.

This pilot study provides information, including sample size calculations for the performance of a RCT. Of the examined patients (N = 96), 50% with NSLBP had at least 3/6 positive MCTs. Because six out of fifty selected patients were excluded from participation because they showed ceiling effects for either the RMQ or the PSFS the future RCT should consider alternative assessments in order to expand the validity of treatment outcomes to a broader range of presenting patients. A limitation of the study is that we did not evaluate the impact of psychosocial risk factors and this analysis is also planned in a future RCT. Dropout rate was low (7%) and adverse effects from testing or treatment were not observed.

Further studies with stricter inclusion criteria should follow. Our study gives preliminary evidence to show that movement control is improvable through specific exercises and gives an indication of a reduction of pain and disability effect. Furthermore, our study has provided data for power analysis calculation for an RCT.

## Conclusions

The results of this controlled case series study, based on prior and post intervention, show that movement control, patient specific functional complaints and disability improved significantly following specific individual exercise programs, performed with physiotherapeutic intervention. Since no control group was included in the study, the results should be treated with some caution. However, the results obtained warrant performance of a further randomized controlled trial (RCT), which is currently planned.

## Competing interests

The authors declare that they have no competing interests.

## Authors' contributions

HL accumulated the data, calculated statistics, and was the main author of the paper. JK was involved in the planning of the study, methodological considerations, analysis of the data, and critically revised the manuscript for its content.

EdB was involved in the planning of the study, methodological considerations, and critically revised the manuscript for its content.

OA was involved in the planning of the study, methodological considerations, and revised the paper. All authors read and approved the final manuscript.

## References

[B1] Waddell G Back pain revolution 2004 2 Churchill-Livingstone;

[B2] Airaksinen O Brox J Cedraschi C Hildebrandt J Klaber-Moffett J Kovacs F Chapter 4. European guidelines for the management of chronic nonspecific low back pain Eur Spine J 2006 15 Suppl 2 S192 300 10.1007/s00586-006-1072-1 16550448 PMC3454542

[B3] Borkan J Van Tulder M Reis S Schoene ML Croft P Hermoni D Advances in the field of low back pain in primary care: a report from the fourth international forum Spine (Phila Pa 1976) 2002 27 5 E128 32 11880849 10.1097/00007632-200203010-00019

[B4] van Tulder M Koes B Bombardier C Low back pain Best Pract Res Clin Rheumatol 2002 16 5 761 75 10.1053/berh.2002.0267 12473272

[B5] Pengel L Herbert R Maher C Refshauge K Acute low back pain: systematic review of its prognosis BMJ 2003 327 7410 323 10.1136/bmj.327.7410.323 12907487 PMC169642

[B6] van Tulder M Koes B Low back pain and sciatica: acute Clin Evid 2002 7 1018 31 12230724

[B7] van Tulder M Koes B Low back pain and sciatica: chronic Clin Evid 2002 7 1032 48 12230725

[B8] Dankaerts W O'Sullivan PB Burnett AF Straker LM The use of a mechanism-based classification system to evaluate and direct management of a patient with non-specific chronic low back pain and motor control impairment--A case report Manual Therapy 2007 in press 16877024 10.1016/j.math.2006.05.004

[B9] O'Sullivan PB Masterclass. Lumbar segmental 'instability': clinical presentation and specific stabilizing exercise management Manual Therapy 2000 5 1 2 12 10.1054/math.1999.0213 10688954

[B10] O'Sullivan P Diagnosis and classification of chronic low back pain disorders: Maladaptive movement and motor control impairments as underlying mechanism Manual Therapy 2005 10 4 242 55 10.1016/j.math.2005.07.001 16154380

[B11] Vibe Fersum K O'Sullivan PB Kvale A Skouen JS Inter-examiner reliability of a classification system for patients with non-specific low back pain Man Ther 2009 14 5 555 61 10.1016/j.math.2008.08.003 18838331

[B12] Van Dillen LR Sahrmann SA Norton BJ Caldwell CA McDonnell MK Bloom NJ Movement system impairment-based categories for low back pain: stage 1 validation The Journal of orthopaedic and sports physical therapy 2003 33 3 126 42 12683688 10.2519/jospt.2003.33.3.126

[B13] Sahrmann SA Diagnosis and treatment of movement impairment syndromes Anonymous 2002 St.Louis: Mosby

[B14] Comerford MJ Mottram SL Functional stability re-training: principles and strategies for managing mechanical dysfunction Manual Therapy 2001 6 1 3 14 10.1054/math.2000.0389 11243904

[B15] Comerford MJ Mottram SL Movement and stability dysfunction - contemporary developments Manual Therapy 2001 6 1 15 26 10.1054/math.2000.0388 11243905

[B16] Maluf KS Sahrmann SA Van Dillen LR Use of a classification system to guide nonsurgical management of a patient with chronic low back pain Physical Therapy 2000 80 11 1097 111 11046197

[B17] Van Dillen LR Sahrmann SA Wagner JM Classification, intervention, and outcomes for a person with lumbar rotation with flexion syndrome Physical Therapy 2005 85 4 336 51 15794704

[B18] Dankaerts W O'Sullivan PB Straker LM Burnett AF Skouen JS The inter-examiner reliability of a classification method for non-specific chronic low back pain patients with motor control impairment Manual therapy 2006 11 1 28 39 10.1016/j.math.2005.02.001 15936976

[B19] Van Dillen LR Sahrmann SA Norton BJ Caldwell CA Fleming DA McDonnell MK Reliability of physical examination items used for classification of patients with low back pain Physical Therapy 1998 78 9 979 88 9736895 10.1093/ptj/78.9.979

[B20] Luomajoki H Kool J de Bruin E Airaksinen O Reliability of movement control tests in the lumbar spine BMC Musculoskelet Disord 2007 8 90 10.1186/1471-2474-8-90 17850669 PMC2164955

[B21] Moseley G Zalucki N Birklein F Marinus J van Hilten J Luomajoki H Thinking about movement hurts: the effect of motor imagery on pain and swelling in people with chronic arm pain Arthritis Rheum 2008 59 5 623 31 10.1002/art.23580 18438892

[B22] Van Dillen LR Sahrmann SA Norton BJ Caldwell CA McDonnell MK Bloom N The effect of modifying patient-preferred spinal movement and alignment during symptom testing in patients with low back pain: a preliminary report Archives of Physical Medicine and Rehabilitation 2003 84 3 313 22 10.1053/apmr.2003.50010 12638097

[B23] Murphy DR Byfield D McCarthy P Humphreys K Gregory AA Rochon R Interexaminer reliability of the hip extension test for suspected impaired motor control of the lumbar spine Journal of manipulative and physiological therapeutics 2006 29 5 374 7 10.1016/j.jmpt.2006.04.012 16762665

[B24] White LJ Thomas ST The rater reliability of assessments of symptom provocation in patients with low back pain Journal of Back and Musculoskeletal Rehabilitation 2002 16 83 90 10.3233/bmr-2002-162-306 22387404

[B25] Hayden JA van Tulder MW Malmivaara A Koes BW Exercise therapy for treatment of non-specific low back pain Cochrane Database Syst Rev 2005 3 CD000335 16034851 10.1002/14651858.CD000335.pub2 PMC10068907

[B26] Hayden JA van Tulder MW Tomlinson G Systematic review: strategies for using exercise therapy to improve outcomes in chronic low back pain Ann Intern Med 2005 142 9 776 85 15867410 10.7326/0003-4819-142-9-200505030-00014

[B27] van Tulder MW Croft PR van Splunteren P Miedema HS Underwood MR Hendriks HJ Disseminating and implementing the results of back pain research in primary care Spine (Phila Pa 1976) 2002 27 5 E121 7 11880848 10.1097/00007632-200203010-00018

[B28] Hayden J Multiple choices for certification Health Promot Pract 2005 6 3 258 62 10.1177/1524839905275370 16106567

[B29] van Tulder MW Ostelo R Vlaeyen JW Linton SJ Morley SJ Assendelft WJ Behavioral treatment for chronic low back pain: a systematic review within the framework of the Cochrane Back Review Group Spine (Phila Pa 1976) 2000 25 20 2688 99 11034658 10.1097/00007632-200010150-00024

[B30] O'Sullivan PB Phyty GD Twomey LT Allison GT Evaluation of specific stabilizing exercise in the treatment of chronic low back pain with radiologic diagnosis of spondylolysis or spondylolisthesis Spine (Phila Pa 1976) 1997 22 24 2959 67 9431633 10.1097/00007632-199712150-00020

[B31] Stuge B Laerum E Kirkesola G Vollestad N The efficacy of a treatment program focusing on specific stabilizing exercises for pelvic girdle pain after pregnancy: a randomized controlled trial Spine (Phila Pa 1976) 2004 29 4 351 9 15094530 10.1097/01.brs.0000090827.16926.1d

[B32] Luomajoki H Kool J de Bruin ED Airaksinen O Movement control tests of the low back; evaluation of the difference between patients with low back pain and healthy controls BMC Musculoskelet Disord 2008 9 170 10.1186/1471-2474-9-170 19108735 PMC2635372

[B33] Luomajoki H Kool J de Bruin ED Airaksinen O Reliability of movement control tests in the lumbar spine BMC Musculoskelet Disord 2007 8 90 10.1186/1471-2474-8-90 17850669 PMC2164955

[B34] Stradford PGC Westway M Binkley J Assessing disability and change on individual patients: A report of a patient specific measure Physiotherapy canada 1995 47 4 258 63 10.3138/ptc.47.4.258

[B35] Roland M Morris R A study of the natural history of low-back pain. Part II: development of guidelines for trials of treatment in primary care Spine (Phila Pa 1976) 1983 8 2 145 50 6222487 10.1097/00007632-198303000-00005

[B36] Roland M Morris R A study of the natural history of back pain. Part I: development of a reliable and sensitive measure of disability in low-back pain Spine (Phila Pa 1976) 1983 8 2 141 4 6222486 10.1097/00007632-198303000-00004

[B37] Beurskens A de Vet H Köke A Heijden G van der Knipschild P Measuring the functional status of patients with low back pain. Assessment of the quality of four disease-specific questionnaires Spine 1995 20 9 1017 28 10.1097/00007632-199505000-00008 7631231

[B38] Beurskens A de Vet H Köke A Responsiveness of functional status in low back pain: a comparison of different instruments Pain 1996 65 1 71 6 10.1016/0304-3959(95)00149-2 8826492

[B39] Deyo R Battie M Beurskens A Bombardier C Croft P Koes B Outcome measures for low back pain research. A proposal for standardized use Spine 1998 23 18 2003 13 10.1097/00007632-199809150-00018 9779535

[B40] Pengel L Refshauge K Maher C Responsiveness of pain, disability, and physical impairment outcomes in patients with low back pain Spine 2004 29 8 879 83 10.1097/00007632-200404150-00011 15082988

[B41] Stratford P Binkley J Solomon P Finch E Gill C Moreland J Defining the minimum level of detectable change for the Roland-Morris questionnaire Phys Ther 1996 76 4 359 65 discussion 66-8. 8606899 10.1093/ptj/76.4.359

[B42] Cook C Brismee JM Sizer PS Jr Subjective and objective descriptors of clinical lumbar spine instability: a Delphi study Manual therapy 2006 11 1 11 21 10.1016/j.math.2005.01.002 15996889

[B43] van Tulder M Malmivaara A Esmail R Koes B Exercise therapy for low back pain: a systematic review within the framework of the cochrane collaboration back review group Spine (Phila Pa 1976) 2000 25 21 2784 96 11064524 10.1097/00007632-200011010-00011

[B44] Kool J de Bie R Oesch P Knusel O Brandt P van den Bachmann S Exercise reduces sick leave in patients with non-acute non-specific low back pain: a meta-analysis J Rehabil Med 2004 36 2 49 62 10.1080/16501970310020104 15180219

[B45] Kool J Bachmann S Oesch P Knuesel O Ambergen T de Bie R Function-centered rehabilitation increases work days in patients with nonacute nonspecific low back pain: 1-year results from a randomized controlled trial Arch Phys Med Rehabil 2007 88 9 1089 94 10.1016/j.apmr.2007.05.022 17826451

[B46] Kool JP Oesch PR Bachmann S Knuesel O Dierkes JG Russo M Increasing days at work using function-centered rehabilitation in nonacute nonspecific low back pain: a randomized controlled trial Arch Phys Med Rehabil 2005 86 5 857 64 10.1016/j.apmr.2004.10.044 15895328

[B47] Rackwitz B de Bie R Limm H von Garnier K Ewert T Stucki G Segmental stabilizing exercises and low back pain. What is the evidence? A systematic review of randomized controlled trials Clin Rehabil 2006 20 7 553 67 10.1191/0269215506cr977oa 16894798

[B48] Costa LO Costa Lda C Cancado RL Oliveira Wde M Ferreira PH Short report: intra-tester reliability of two clinical tests of transversus abdominis muscle recruitment Physiotherapy Research International: The Journal for Researchers and Clinicians in Physical Therapy; Physiotherapy Research International: The Journal for Researchers and Clinicians in Physical Therapy 2006 11 1 48 50 10.1002/pri.39 16594315

[B49] Stuge B Veierod MB Laerum E Vollestad N The efficacy of a treatment program focusing on specific stabilizing exercises for pelvic girdle pain after pregnancy: a two-year follow-up of a randomized clinical trial Spine (Phila Pa 1976) 2004 29 10 E197 203 15131454 10.1097/00007632-200405150-00021

[B50] Brennan GP Fritz JM Hunter SJ Thackeray A Delitto A Erhard RE Identifying subgroups of patients with acute/subacute "nonspecific" low back pain: results of a randomized clinical trial Spine 2006 31 6 623 31 10.1097/01.brs.0000202807.72292.a8 16540864

[B51] Concato J Shah N Horwitz R Randomized, controlled trials, observational studies, and the hierarchy of research designs N Engl J Med 2000 342 25 1887 92 10.1056/NEJM200006223422507 10861325 PMC1557642

[B52] Kendall NLS Main C Guide to assessing psychosocial yellow flags in acute low back pain 1997 Wellington, New Zealand

[B53] Linton SJ Andersson T Can chronic disability be prevented? A randomized trial of a cognitive-behavior intervention and two forms of information for patients with spinal pain Spine (Phila Pa 1976) 2000 25 21 2825 31 discussion 4. 11064530 10.1097/00007632-200011010-00017

[B54] Vlaeyen JW Linton SJ Fear-avoidance and its consequences in chronic musculoskeletal pain: a state of the art Pain 2000 85 3 317 32 10.1016/S0304-3959(99)00242-0 10781906

